# Design and Characterization of Sodium Alginate and Poly(vinyl) Alcohol Hydrogels for Enhanced Skin Delivery of Quercetin

**DOI:** 10.3390/pharmaceutics12121149

**Published:** 2020-11-27

**Authors:** Ludovico Esposito, Ana Isabel Barbosa, Tânia Moniz, Sofia Costa Lima, Paulo Costa, Christian Celia, Salette Reis

**Affiliations:** 1 LAQV, REQUIMTE, Departamento de Ciências Químicas, Faculdade de Farmácia, Universidade do Porto, Rua de Jorge Viterbo Ferreira, 228, 4050-313 Porto, Portugal; ludovico.esposito@studenti.unich.it (L.E.); up200800307@ff.up.pt (A.I.B.); tmoniz@ff.up.pt (T.M.); shreis@ff.up.pt (S.R.); 2 Department of Pharmacy, University “G.d’Annunzio” Chieti-Pescara, 66013 Chieti, Italy; c.celia@unich.it; 3 UCIBIO, REQUIMTE, MedTech, Departamento de Ciências do Medicamento, Faculdade de Farmácia, Universidade do Porto, Rua de Jorge Viterbo Ferreira, 228, 4050-313 Porto, Portugal; pccosta@ff.up.pt

**Keywords:** antioxidant activity, isolated stratum corneum, PVPA*_SC_*, poly(vinyl) alcohol, sodium alginate

## Abstract

Nature has led to the discovery of biopolymers with noteworthy pharmaceutical applications. Blended biopolymers have demonstrated promising characteristics when compared with their individual counterparts. Sodium alginate (SA) is a marine polymer that has demonstrated the ability to form hydrogels, an interesting property for the development of cutaneous formulations. Predicting the good performance of blended biopolymers, a novel series of hybrid hydrogels based on SA and poly(vinyl) alcohol (PVA) were prepared. Quercetin, a natural polyphenolic flavonoid commonly found in fruits and vegetables, is widely known for its strong anti-inflammatory and antioxidant activity, thus with potential applications against melanoma, dermatitis, psoriasis, and skin ageing. Here, hydrogels were produced at different ratios of SA and PVA. The surface morphology, structure, interaction of polymers, the capacity to absorb water and the entrapment efficiency of quercetin were evaluated for the blended hydrogels. Targeting the cutaneous application of the formulations, the rheological properties of all unloaded and quercetin-loaded hydrogels revealed pseudoplastic behavior, evidence of non-thixotropy, good resistance to deformation, and profile maintenance with temperatures ranging from 20 °C up to 40 °C. The incorporation of quercetin in the hydrogel retained its antioxidant activity, confirmed by radical scavenging assays (ABTS and DPPH). The permeability of quercetin through the skin showed different penetration/permeation profiles according to the hydrogel’s blend. This behavior will allow the selection of SA-PVA at 2/1 ratio for a local and prolonged skin effect, making the use of these hydrogels a good solution to consider for the treatment of skin ageing and inflammation.

## 1. Introduction

Hydrogels are hydrophilic polymeric networks with high moisture content, biocompatibility, soft and flexible texture, mechanical properties similar to living tissues, and capacity to alter or control drug release. These features contribute to the growing attention for pharmaceutical and cosmetic applications. Natural and synthetic biopolymers can be used to prepare hydrogels, and usually, hybrid matrices composed of two polymers of both origins resulting in successful balance of the hydrogels’ qualities. In fact, single biopolymer-based hydrogels exhibit poor mechanical properties in the swollen state and are difficult to remove from skin, while artificial polymer-based hydrogels are difficult to optimize in terms of elasticity, stiffness, and hydrophilicity [1]. Alginate is a natural polymer derived from brown algae, chemically composed of 1,4-linked-D-mannuronic acid (M) and L-guluronic acid (G) residues with capability of forming hydrogels [2]. The hydrophilic nature of alginate as well as its gelation features allow extensive applications for tissue engineering and wound dressing. Yet, limitations arise from poor mechanical properties and shortage of processing. To overcome these, combination of sodium alginate (SA) with a synthetic polymer could produce the optimal properties [3].

Poly(vinyl alcohol) (PVA) is a water soluble synthetic polymer commonly used in drug delivery systems and for tissue engineering, due to its high water content, good biocompatibility, and consistency. A PVA hydrogel usually eases diffusional exchange of its cargo with the external environment, and also exhibits mechanical properties adequate for skin applications. However, PVA hydrogels often have poor elasticity and high stiffness [4]. Thus, the design of a SA-PVA hybrid hydrogel could contribute to the preparation of a thin gel optimized for transdermal drug delivery, wound dressings, or cosmetic applications. In fact, hydrogels have been gaining interest among research groups for cutaneous application instead of other conventional dosage forms (creams, pastes, ointments), and the main advantages are related to higher water content and non-greasy texture, better skin feel and cooling effect, better skin hydration and longer drug absorption, reduced transepidermal water loss, skin biocompatibility, and better patient compliance [5,6]. Natural and synthetic polymer blends can form advanced materials with different applications. Blending of polymers produces new materials with optimized properties not achievable with individual polymers [1]. Synthetic polymers can enhance the properties of natural polymers [3]. Here, the role of various amount of each polymer in the swelling and viscoelastic properties are discussed together with transdermal absorption of quercetin to evaluate the potential of a SA-PVA hybrid network in skin applications.

Skin constitutes the first line of defense of the human body against environmental agents, namely pathogens, and ultraviolet radiations among other noxious agents. The accumulation of these stresses in the skin may lead to cutaneous pathologies, like cancer, immune suppression, photoaging, and photocarcinogenesis [7]. Phytochemicals, like quercetin, represent a class of biologically active compounds obtained from plants and herbal products, with proven anti-oxidative, anti-inflammatory, anti-proliferative, and anti-angiogenic effects [7]. Quercetin (2-(3,4-dihydroxyphenyl)-3,5,7-trihydroxy-4Hchromen-4-one) is a polyphenolic compound found in green and black tea, onions, apples, and red grapes. Quercetin acts through scavenging oxygen free-radicals and lipid peroxidation inhibition to exert anti-inflammatory, anti-oxidant, and anti-infective activities [8]. These are beneficial in managing and treating several cutaneous pathologies. Yet, quercetin presents limited capability to penetrate the skin due to the polar hydroxyl groups, and is also hampered by its poor water solubility (ca. 2 µg/mL) [9] and, light, temperature, and pH instability. To reach therapeutic levels in the organism, quercetin requires a carrier with high loading capacity and good adherence to the skin. Besides the drawbacks associated with the physicochemical properties of quercetin, another major hindrance is the *stratum corneum* (*SC*). This outer layer hampers drug absorption through the skin resulting in low bioavailability. To overcome these issues, drug delivery systems have been designed for quercetin cutaneous transport based on nanocarrier systems as liposomes [10,11], self-polymerization of quercetin [12], mannosylated liposomes [13], nanogels [14,15], deformable liposomes [16], niosomes [17], microemulsions [18], thermo-responsive mesoporous silica [19], polymeric nanoparticles [20], and hydrogels [21] which can protect the skin from oxidation, inflammation, and photoaging, and reinforce the immune system during wound healing process. Among the most promising approaches, hydrogels appear as an attractive option, given their skin adhesiveness and hydration abilities.

Based on this evidence, the current study aimed to determine which combination of SA-PVA, considering different polymer ratios (1/1, 2/1, or 1/2), delivered quercetin to the skin, thus increasing its local bioavailability and maintaining its antioxidant activity. Quercetin was formulated in SA-PVA hybrid hydrogels, characterized in terms of surface morphology, structure, and interaction of polymers and water absorbing capacity, and evaluated in a skin permeation model. The resulting hybrid and therapeutic hydrogels could represent a potential topical formulation for the treatment of skin ageing and inflammation.

## 2. Materials and Methods

### 2.1. Materials and Instrumentation

Sodium alginate was purchased from ACROS Organics^TM^ (Thermo Fisher Scientific, Waltham, MA, USA). Poly(vinyl alcohol); quercetin (2-(3,4-dihydroxyphenyl)-3,5,7-trihydroxy-4Hchromen-4-one); 2,2′-azinobis-(3-ethylbenzothiazoline-6-sulphonate) (ABTS); potassium persulfate; 2,2-diphenyl-1-picrylhydrazyl radical (DPPH); methanol; L-α-phosphatidylcholine (EPC); dimethylsulfoxide (DMSO); Dulbecco’s phosphate buffered saline (DPBS) (10×); ethanol (absolute); sodium cholesteryl sulphate; trypsin (from porcine pancreas); and the Millicell Cell Culture Inserts (12 mm, polycarbonate, 0.4 µm) were purchased from Sigma-Aldrich (St. Louis, MO, USA). Cholesterol (ovine wool) and egg chicken ceramide and were obtained from Avanti Polar Lipids (Alabaster, AP, USA). The porcine ears were acquired in a local slaughterhouse located in Porto, Portugal. The ultra-pure water system (Arium Pro, Sartorius AG, Gottingen, Germany) was the source of double-deionized water. Reagents were weighted in a Kern ACJ/ACS 80-4 (Kern & Sohn; Balingen, Germany) digital analytical balance, and a Crison pH meter GLP 22 with a Crison 52-02 tip (Crison; Barcelona, Spain) was used to perform the necessary pH measurements.

### 2.2. Methods

#### 2.2.1. SA-PVA Hydrogel Preparation

For hydrogel preparation, SA was dissolved in double-deionized water (7.5% *w/v*) using a glass rod. After complete dissolution, a previously prepared PVA solution (8% *w/v* in double-deionized water) was added to the SA and mixed again with the glass rod, according to different SA-PVA ratios (1/1, 2/1, and 1/2). The mixing with a glass rod was an in-house developed method which allowed a better incorporation of the synthetic polymer in the natural polymer solution. In quercetin-loaded hydrogels, 1% (*w/w*) of quercetin was added to the hydrogel blend in the SA dissolution step. After complete hydrogel preparation, the quercetin concentration was 0.8 mg mL^−1^. To remove the air bubbles from hydrogel mixing, all hydrogels were left degassing in an incubator shaker (ES-60E Incubator Shaker, Miulab, Hangzhou, Zhejiang, China) at room temperature and 125 rpm, for 48 h.

#### 2.2.2. Physicochemical Characterization of the SA-PVA Hydrogels

The hydrogels were analyzed using a FTIR Spectrophotometer (Frontier^TM^, PerkinElmer; Santa Clara, CA, USA) equipped with a diamond crystal, after lyophilization process. To do so, all hydrogels were kept overnight in a −80 °C freezer (Deep Freezer, GFL^®^, Burgwedel, Germany), to be further lyophilized in a freeze drier (LyoQuest –85 plus v.407, Telstar^®^ Life Science Solutions, Terrassa, Spain) for 72 h, continuously kept at −80 °C and 0.40 mbar of pressure. The empty and quercetin-loaded lyophilized hydrogels, as well as all reference compounds, were placed directly into the ATR compartment for analysis at room temperature, after a background run with empty ATR accessory to be considered as a negative control. Obtained spectra were a result of 32 combined scans recorded between 4000 and 600 cm^−1^, with spectral resolution of 4 cm^−1^.

The freeze-dried hydrogels were analyzed by scanning electron microscopy (SEM) using a FEI Quanta 400 FEG ESEM/EDAX Pegasus X4M with an accelerating voltage of 10 kV. Hydrogels were fixed onto carbon-taped metal pins and coated with Au/Pd by sputtering for 45 s.

#### 2.2.3. Determination of Quercetin Loading in the SA-PVA Hydrogels

For quercetin quantification, 100 mg of each quercetin-loaded hydrogel was dissolved in 2 mL of DMSO, followed by 2 min of vortex and 45 min of ultrasound bath, to destroy the hydrogel matrix and release the compound. The samples were then centrifuged for 15 min at 10,000× *g* using an Allegra X-15R centrifuge (Beckman Coulter, Pasadena, CA, USA), in order to pellet the destroyed hydrogel. To confirm that all quercetin was released to the supernatant, a second extraction was performed, dissolving the hydrogel pellet in 2 mL of fresh DMSO, followed by the same process of vortex, ultrasound bath, and centrifugation. The amount of quercetin in the supernatants was quantified using an ultraviolet–visible light (UV–Vis) spectrophotometer (Jasco V-660 Spectrophotometer, Software: Spectra Manager v.2, Jasco Corporation, Easton, Maryland, USA) at 370 nm. A calibration curve (absorbance = 0.0634 (quercetin) + 0.015) was obtained in DMSO for the concentration range 0 to 40 µg mL^−1^ of quercetin, with a R^2^ of 0.9991.

#### 2.2.4. Swelling Assay

The swelling ratio (SR) of hydrogels was studied in double-deionized water by measuring the mass weight after swollen at different time points (1, 2, and 3 h). Three grams of all hydrogels were placed in 40 mL of double-deionized water, in an incubator shaker (ES-60E Incubator Shaker, Miulab, Hangzhou, Zhejiang, China) at 32 °C and 125 rpm. Samples were then carefully withdrawn, removing all free water, and then weighted. The data are expressed as the mean ± standard deviation (SD) of three independent experiments, calculating SR (*w/w*) according to the following equation:

(1) $$\mathrm{SR}=\frac{W_{wet}-W_{dry}}{W_{dry}}$$

#### 2.2.5. Rheology Studies

The rheological properties of the prepared hydrogels were analyzed on a rheometer (Malvern Kinexus Lab+; Malvern Instruments; Worcestershire, UK) using four different methods. For viscosimetry, a shear rate table method (0.1 to 100.0 s^−1^, 10 samples per decade, 25 °C) was used. The thixotropy test followed a three-step shear rate method (1st phase: 0.1 s^−1^, 2 min; 2nd phase: 100.0 s^−1^, 30 s; 3rd phase: 0.1 s^−1^, 15 min, 25 °C). To determine linear viscoelastic region, an amplitude sweep method was performed (0.1 to 100%, 10 samples per decade, 1.0 Hz, 25 °C). Finally, to address the temperature effect, a single frequency temperature ramp was used (initial temperature 20 °C, final temperature 40 °C, 1 °C/min ramp, frequency 1 Hz). All analysis was conducted with a plate-plate configuration (geometry PU20 SR4367) with a 1 mm gap (Peltier Plate Cartridge). The data were collected using the rSpace software^®^ (Kinexus 1.75: PSS0211-17).

#### 2.2.6. Evaluation of the Antioxidant Activity

The 2,2′-azinobis-(3-ethylbenzothiazoline-6-sulphonate) radical cation decolorization test (ABTS assay) was used to analyze the antioxidant activity of unloaded and quercetin-loaded hydrogels. The ABTS solution was prepared mixing equal volumes of 7 mM ABTS and 2.45 mM potassium persulfate, separately prepared in water, and left incubating overnight at room temperature protected from light. For sample analysis, the ABTS solution was diluted with the double deionized water to an absorbance of 0.90 ± 0.02 at 734 nm, and 100 µL of diluted hydrogels (1 to 5 mg mL^−1^) and free quercetin solutions were added to 100 µL of diluted ABTS solution. After 15 min of incubation protected from light, Synergy^TM^ HT Multimode plate reader (BioTek^®^ Instruments Inc., Winooski, VT, USA) was used to measure sample absorbance (*A_Sample_*). A sample blank (*A_Control_*) was tested in each assay, all determinations were carried out in triplicate, and the percentage of radical scavenging activity (% *RSA*) was determined using the equation:

(2) $$RSA\;\left(\%\right)=\frac{A_{Control}-A_{Sample}}{A_{Control}}\times 100$$

The scavenging of 2,2-diphenyl-1-picrylhydrazyl radical (DPPH assay) was also performed to confirm antioxidant activity of all hydrogels. Briefly, 50 mL of 0.2 mM DPPH radical was prepared in methanol and vortexed until complete dissolution. After the addition of 100 µL of DPPH solution to 100 µL of diluted hydrogels (1 to 5 mg mL^−1^) and free quercetin solutions, the absorbance (*A_Sample_*) reading was taken after 15 min. A sample blank (*A_Control_*) was tested in each assay, all determinations were carried out in triplicate, and percentage of radical scavenging activity (% *RSA*) was determined using Equation (2).

#### 2.2.7. Permeation Assays

##### Quercetin Permeation Assay through Isolated *SC* Model from Pig Ear Skin

The pig ear skin was used to isolate *SC* layer following the previously reported trypsin digestion method [22,23,24,25,26]. This process involved the immersion and incubation of isolated pig ear skin in trypsin solution (0.1 % *w/v*) for a period of 4 h at 4 °C. The dermis was detached from the *SC* by scraping the skin outside the trypsin solution at defined timepoints, and fresh trypsin solution was used to immerse the scraped skin, allowing it to incubate overnight at 4 °C. The *SC* was further rinsed with ultrapure water to remove the remaining dermis tissue. The *SC* was left to dry in a silica-containing desiccator at atmospheric pressure until completely dried. Integrity of the layer isolated by this procedure was previously reported [26].

The quercetin permeation assays were performed using the dried isolated *SC* portions (circles of approximately 2.5 cm diameter) as model barrier in Franz diffusion cells (9 mm unjacketed Franz Diffusion Cell with 5 mL receptor, O-ring joint, clear glass, clamp, and stir-bar; PermeGear, Inc., Hellertown, PA, USA) [27]. The basolateral compartment was filled with 4.7 mL of PBS (pH 7.4)/10% ethanol and 500 mg of each quercetin-loaded hydrogel was added to the donor chamber. The use of ethanol was previously considered in permeation studies to improve drugs’ solubility in an aqueous medium [26]. The permeation of the free drug was also inspected and for this, the donor chamber was filled with 500 µL (0.65 g L^−1^ of quercetin, in order to mimic the amount of drug loaded in the hydrogels) of solution containing drug dissolved in miglyol. The donor chambers were covered with parafilm. During the study, the Franz cells were stirred, maintained at 32 °C, and protected from light.

For quercetin quantification, sample aliquots of 800 µL were withdrawn from the receptor medium at defined intervals (1, 3, 6, and 24 h) and replaced with equal volumes of the respective fresh buffer. At the last defined interval (24 h), the non-permeated amount of free drug solution or hydrogel in the apical compartment was collected and diluted (25 µL or 25 mg, respectively) in 1 mL of DMSO for further quantification. The percentage of quercetin deposited in the isolated *SC* was determined by calculating the difference between the initial amount of drug and the amount determined in both apical and basolateral compartments [26].

Quercetin concentration was spectrophotometrically determined at 370 nm using a Jasco V-660 Spectrophotometer (Piscataway, NJ, USA). Calibration curves of quercetin in the adequate experimental media were performed and used to assess drug concentration in each assay. For the apical samples, the calibration curve, in DMSO, was absorbance = 0.0389 (quercetin) + 0.1066, R^2^ = 0.9976, for 1–20 µg mL^−1^; while for the basolateral samples, the calibration curve in PBS/10% (*v/v*) ethanol was absorbance = 0.0174 (quercetin) + 0.0843, R^2^ = 0.9911 for 1 to 15 µg mL^−1^. At least three independent experiments were performed. The apparent permeability (*P_app_*) at the 3 h defined timepoint was determined as previously reported [26,28] by estimating the ratio between all quercetin mass (*m_a_*/g) permeated across membranes and the product of the initial mass in apical compartment (*m_d_*/g), the surface area of the *SC* model barrier (*A* = 0.63585 cm^2^), and the time (*t* = 3 h = 10,800 s), as described in the equation:

(3) $$P_{app}\left(\mathrm{cm}/s\right)=\frac{\sum m_{a}}{m_{d}\cdot A\cdot t}$$

##### Quercetin Permeation Assay through PVPA*_SC_*

The PVPA*_SC_* model systems were obtained as previously reported [26]. Briefly, freshly prepared large unilamellar liposomes (LUVs) obtained with L-α-Phosphatidylcholine, ceramide cholesterol, stearic acid, and cholesteryl sulfate were incorporated by two cycles of centrifugation (Allegra X-15R, Beckman Coulter) (950× *g*, 60 min, 20 °C) and one step of incubation at 45 °C for 60 min. Subsequently, the multilamellar large liposomes (MLVs) were added to the insert and centrifuged (1030× *g*, 60 min, 20 °C), followed by a final centrifugation step (20× *g*, 5 min, 20 °C) in an inverted position. Thereafter, the inserts were frozen at −20 °C until used as described elsewhere [26,28].

The quercetin permeation experiments were carried out at 32 °C, with agitation and protection from light. In general, the inserts were placed in a 24-well plate containing 2 mL of PBS (pH 7.4)/10% (v/v) ethanol in the acceptor chamber. Each quercetin-loaded hydrogel was added to the donor chamber in the same amount used for studies considering isolated *SC* models. The permeation of the free drug (quercetin) was also accessed in a similar way. Aliquots of 800 µL were collected at the time points 1, 3, 6, and 24 h and equal quantity of fresh buffer was added.

The quantification of the quercetin concentration in all collected samples and *P_app_* at 3 h was determined as previously described for studies regarding the isolated *SC* model, with exception of the parameter regarding the diffusion area of the inserts (0.60 cm^2^). After 24 h, the remaining free drug solution or hydrogel in the donor chamber was collected and diluted in DMSO for further quantification, as described above.

#### 2.2.8. Statistical Analysis

Statistical analysis of the obtained data was performed using GraphPad Prism Software (Version 6.0 for Windows; GraphPad Software Inc, San Diego, CA, USA). Since the obtained data consist of at least three independent experiments, the results are expressed as mean ± standard deviation (SD), and one-way or two-way analysis of variance (ANOVA) with multiple comparisons were performed, followed by Tukey’s multiple comparison test. A *p* value < 0.05 was considered significant.

## 3. Results and Discussion

### 3.1. Preparation and Characterization of SA-PVA Hybrid Hydrogels

The aim of improving PVA elasticity and stiffness combinations with SA was pursued. Hybrid hydrogels were prepared from various aqueous polymeric blends consisting of SA and PVA, both biocompatible polymers. Three types of hydrogel samples differing in concentration of each polymer were produced to select the best blend composition able to deliver quercetin in the skin. Quercetin was incorporated in the hydrogel matrix by adding 1% (*w/w*) of the flavonoid in each polymeric hydrogel blend. The entrapment efficiency of quercetin-loaded 1/1, 2/1, and 1/2 hydrogels was determined for three different batches of each hybrid hydrogel, revealing an incorporation efficiency of (80 ± 7)%, (94 ± 15)%, and (88 ± 1)%, respectively. The similarity observed in the incorporation efficient values is expected as equivalent amounts of polymers were used for each blend.

**Figure 1:**
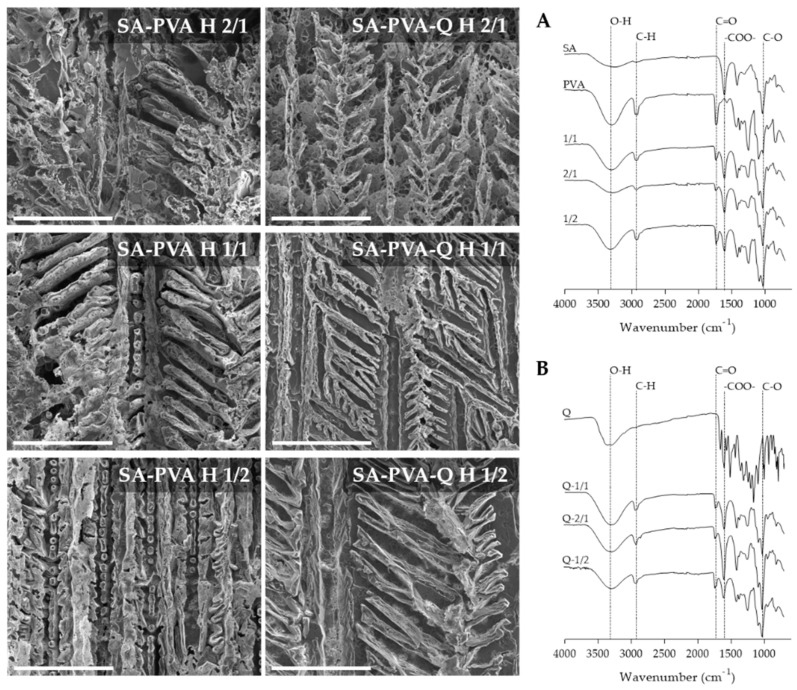
Analysis of the hydrogels morphology (left) and chemical interactions (right). SEM micrographs of quercetin free and quercetin-loaded sodium alginate-poly(vinyl) alcohol (SA-PVA) hydrogels. Scale bar 200 μm. FTIR spectra of empty (**A**) and quercetin-loaded (**B**) hydrogels.

SEM micrographs of quercetin free and quercetin-loaded SA-PVA at different ratios depicting the morphology, distribution, and alignment are shown in Figure 1. SA-PVA bulk gel showed an interconnected branched structure. Data obtained suggested an increase in branching with the incorporation of quercetin and the formation of micropores more evident in the 2/1 and 1/2 hybrid hydrogels. Therefore, it can be concluded that the presence of quercetin may affect the hydrogel matrix. Considering the potential application of quercetin-loaded SA-PVA hydrogel on skin applications, the pores may improve the release of the active substances from the material in a controlled manner. Chen and collaborators working with SA-PVA observed similar branched structures of porous [29].

FTIR is an analytical technique that identifies the functional groups in organic, polymeric, and inorganic materials, and was applied to identify the functional groups in the developed hydrogels based on sodium alginate and PVA molecules. The characteristic IR spectrum of PVA exhibits absorption of C–OH at 1050–1150 cm^−1^, O–H stretching at 3200–3600 cm^−1^, CH_2_ twisting vibration around 1023 cm^−1^, and C–H stretching of CH_2_ group at 2850–3000 cm^−1^ [30]. Figure 1A,B summarizes the characteristic sodium alginate absorption bands at around 1574 cm^−1^ and 1416 cm^−1^, related to the asymmetric and symmetric stretching modes of the carboxylate anion. The stretching vibrations of CO–C group (1032 cm^−1^) are also present. The IR spectra of the hydrogels obtained with different ratios of SA and PVA show the peaks described for pure SA and PVA, which indicates the formation of blends based on both moieties. The IR spectral analysis indicates the synthesis of hybrid hydrogels of SA blend with PVA. Effective bends of polymers in the hydrogels were also verified by FTIR with other combinations [4].

The IR spectrum of free quercetin shows characteristic bands of 1381 cm^−1^ (C–OH), 1610 cm^−1^ (C=C), 1264 cm^−1^ (C–O–C), 1662 cm^−1^ (C=O), and 3403 cm^−1^ (O–H stretch). Yet, these typical molecular peaks of quercetin are not observed in the spectrum of quercetin-loaded hydrogels, which may indicate that the incorporation depended on the interactions mediated by hydrophobic interaction or hydrogen bonds. Reports of quercetin loaded in nanoparticles also describe the disappearance of quercetin characteristic peaks upon incorporation of quercetin [31,32].

### 3.2. Swelling Characteristics

**Figure 2:**
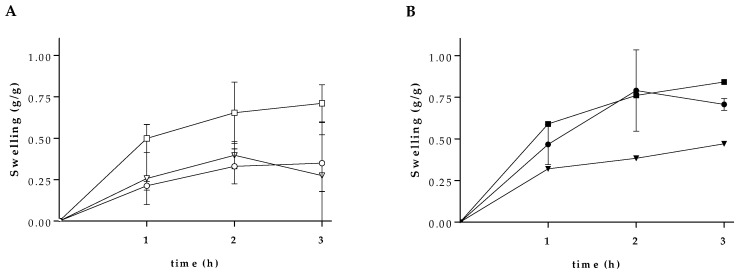
Swelling ratio of SA-PVA hydrogels. 1/1 (circle), 2/1 (square), and 1/2 (inverted triangle) empty hydrogels (**A**) and quercetin-loaded hydrogels (**B**) were soaked in water up to 3 h.

The evaluation of the swelling profile of hydrogels gives important information regarding the bioactive molecule release patterns from these polymeric networks. The rate and degree of swelling are related to crosslinking and mechanical strength of the hydrogels, and depend on the medium (e.g., salts, acids, and bases). The SA-PVA hydrogels were assessed for swelling profile, by measuring the weight of swelled hydrogels at different time, as shown in Figure 2. The maximum swelling ability changes in dependence on the SA ratio (Figure 2A). These findings are in line with previous studies where alginate-based materials exhibit good swelling properties [33,34]. Each blend was then loaded with quercetin and the loaded hydrogels were then evaluated for the swelling effect. As shown in Figure 2B, the presence of quercetin improved the swelling capacity of the 1/1 hydrogel and produced non-significant effect on the SA-PVA hydrogel 2/1 and 1/2. The 1/1 SA-PVA hydrogel contains equal amount of each polymer in relation to the 2/1 and 1/2 blends, which may favor the interaction of quercetin with the polymers and allow absorption of higher amounts of water [34].

### 3.3. Rheological Analysis of SA-PVA Hydrogels

Rheological analysis has been a useful tool in the development and characterization of hydrogel systems for cutaneous application [35]. Following several studies, rheology of polymeric hydrogels has led to a better understanding of the interaction behind the polymers, and their mechanical and flow properties, particularly as a way to compare them to other commercially available hydrogel formulations [36]. Aiming for a cutaneous application and an accurate perception of how the designed formulations will flow through skin, all empty and quercetin-loaded hydrogels were submitted to rheology studies, performing viscosimetry, thixotropy, and resistance to deformation and temperature.

**Figure 3:**
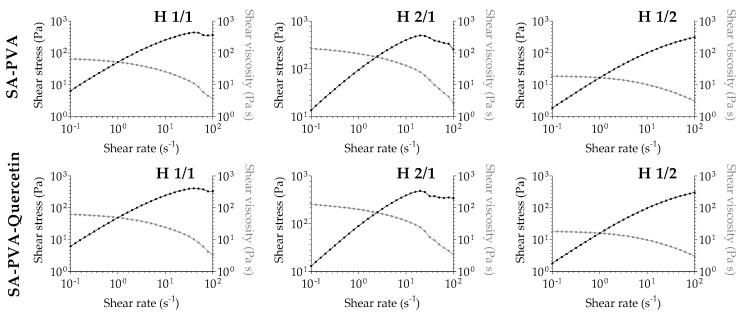
Viscosimetry analysis of unloaded (top row) and quercetin-loaded (bottom row) SA-PVA hydrogels, through shear stress (black line) and shear viscosity (grey line) data.

Considering the viscosimetry evaluation (Figure 3), the first drawn conclusion states that the higher amount of SA in the hydrogel ratio leads to a higher viscosity, in a way that the increasing viscosity follows the order 1/2 < 1/1 < 2/1. The same rule applies to the corresponding quercetin-loaded formulations, confirming that the flavonoid incorporation did not affect the hydrogel structure and characteristics. This results in graphical information that is practically superimposable for unloaded and corresponding quercetin-loaded formulations. It is also possible to find a shear thinning behavior in all unloaded and quercetin-loaded combinations: shear stress increases and shear viscosity decreases with increasing shear rate, indicating a pseudoplastic behavior. Pseudoplasticity is observed as a decrease in the viscosity of the gel with increasing shear rate, and it is important for topical hydrogel formulations that are viscous under static conditions, but become less viscous after application of shear stress, resulting in better spreadability and improved drug permeation of the active substances upon cutaneous application [37,38].

Thixotropy is generally recognized as the phenomenon by which a viscous structure turns less viscous/liquid over time under the same shear rate, being a reversible effect over time when left at rest to rebuild the initial conformation [37]. Therefore, this phenomenon has been widely explored in pharmaceutical applications, particularly in hydrogels, sunscreens, and other topical formulations [37]. In this study, all hydrogels were found to be non-thixotropic.

**Figure 4:**
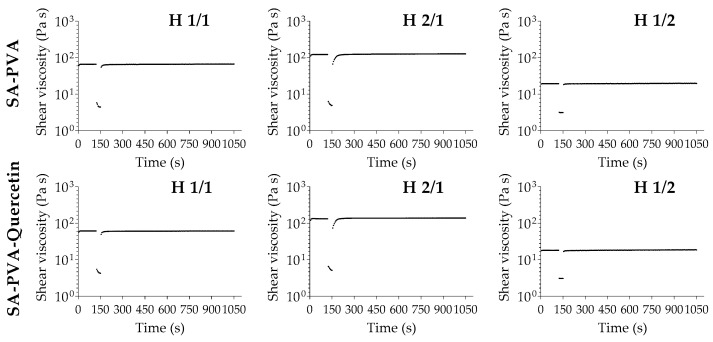
Thixotropy analysis of unloaded (top row) and quercetin-loaded (bottom row) SA-PVA hydrogels.

In Figure 4, the initial viscosity of the hydrogels for the first two minutes is represented, then a high shear was applied to the sample for thirty seconds to induce extreme stress conditions, and finally, the viscosity was monitored for fifteen minutes to follow its recovery. As is visible in all hydrogels, the initial viscosity was almost instantaneously regained with a rebuild time of approximately 20 s, with complete recovery of the initial viscosity conditions, confirming the non-thixotropic profile. This profile also suggests that all hydrogels present self-healing capacity to re-establish their original state spontaneously through time after stress, a characteristic that was also reported for other SA-PVA hydrogels [39].

**Figure 5:**
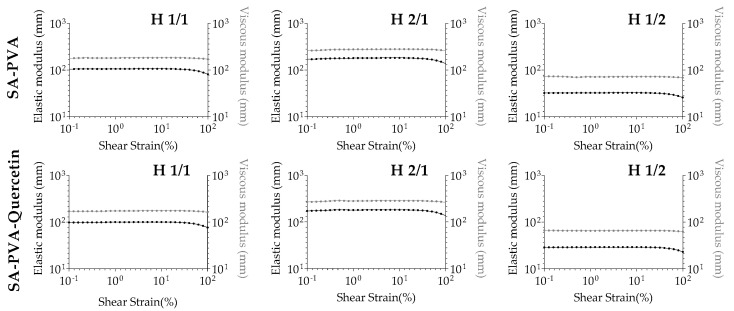
Resistance to deformation from determination of viscoelastic region of unloaded (top row) and quercetin-loaded (bottom row) SA-PVA hydrogels.

The resistance to deformation was also assessed in this study. Since cutaneous application implies a stress application to perform the spread-out capability of formulations, there is the possibility of polymeric matrix rearrangement, resulting in the destruction of the hydrogel characteristics [40]. Performing an amplitude sweep test and maintaining room temperature of 25 °C, the hydrogels were submitted to different amplitudes to determine the linear viscoelastic region (Figure 5). The constant profile of both elastic and viscous modulus in all preparations indicates that deformation only occurred from values higher than 90% of shear strain. The hydrogels can be classified as the equivalent of an apparent viscous liquid with high resistance to deformation, confirming the good stability of both unloaded and quercetin-loaded formulations.

The constant profile maintenance of elastic and viscous modulus was also confirmed by good resistance to a temperature increase from 20 to 40 °C (Figure S1). This feature is important not only to predict the behavior of the hydrogels in contact with the skin (approximately at 32 °C), but also to determine storage stability conditions. The viscosity reduction due to temperature increase has already been described for other hydrogels, this being a general effect in all tested formulations [41]. From all formulations, the 1/2 ratio and corresponding quercetin-loaded hydrogel suffered the most relevant changes in terms of elasticity and viscosity, altering to a more liquid-like form.

The overall rheology study revealed robust interpenetrating polymer network hydrogels, whose initial characteristics were not negatively affected by the incorporation of quercetin in its matrix, with pseudoplastic behavior, non-thixotropic profile, and good resistance to deformation and temperatures from 20 to 40 °C, making them good candidates for cutaneous application.

### 3.4. Antioxidant Activity of Quercetin Incorporated within SA-PVA Hydrogels

**Figure 6:**
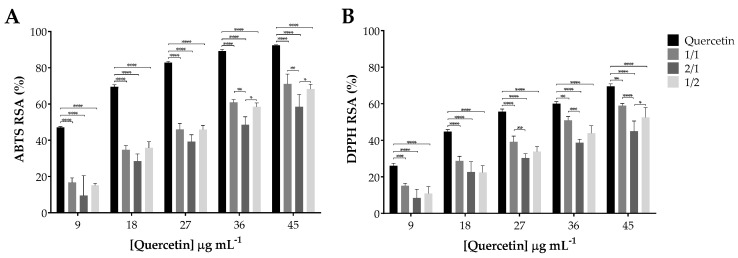
Antioxidant activity of quercetin incorporated within SA-PVA hydrogels. (**A**) ABTS and (**B**) DPPH radical scavenging activity percentage of free quercetin and quercetin-loaded SA-PVA hydrogels. Data points correspond to mean ± standard deviation for *n* = 3 replicates; * *p* < 0.05, ** *p* < 0.01, *** *p* < 0.001, **** *p* < 0.0001.

The antioxidant activity of quercetin plays a major role in its therapeutic effect. Hence, it is fundamental to verify that the hydrogel system does not interfere with this inherent activity of the cargo. The ABTS and the DPPH scavenging assays are widely used methods for the assessment of the antioxidant capacities of natural products, through spectrophotometric techniques. Both assays are based on quenching of stable colored radicals (ABTS or DPPH) and revealing the radical scavenging ability of antioxidants. Figure 6 presents the results obtained and reveals that the scavenging activity of quercetin, both free and incorporated within the SA-PVA hydrogels, increased with its concentration. Upon incorporation in the SA-PVA hydrogels, the antioxidant activity of quercetin decreased in relation to its free form in ca. 20% and 10% for the ABTS (Figure 6A) and DPPH (Figure 6B) scavenging assay, respectively. Among the three blends used for the SA-PVA hydrogels, and considering all ranges of concentrations in both tests, no significant statistical differences (*p* > 0.05) were found between quercetin-loaded SA-PVA 1/1 and 1/2. Particularly in the highest tested concentrations, the quercetin-loaded SA-PVA 2/1 hydrogel manifested the lowest antioxidant capacity, being statistically different from the other two hydrogel blends, probably related to a higher polymeric matrix protection of quercetin. In fact, quercetin’s antioxidant activity is higher for the quercetin-loaded 1/1 and 1/2 hydrogels.

### 3.5. Permeation of Quercetin-Loaded Hydrogels in SC Models

The permeation of the quercetin-loaded hydrogels was evaluated considering the ex vivo skin mimetic models PVPA*_SC_* barrier and isolated *SC* layer obtained from pig ear skin. The results obtained using the isolated *SC* models presented some data variability, as shown by the high standard deviation values obtained from the reported results (Figure S2). Similar findings were recently reported [26]. Thus, alternative skin mimetic models, as the PVPA*_SC_* barriers, were considered to overcome thus improve data reproductivity [42].

**Figure 7:**
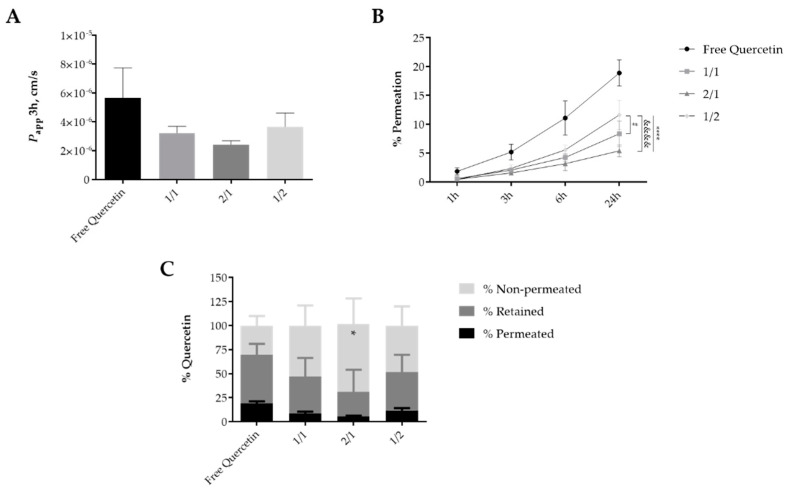
Quercetin permeation profile through the PVPA*_SC_* barrier. (**A**) Apparent permeability (*P_app_*) of quercetin at 3 h; (**B**) Amount of permeated quercetin (%) as a function of time obtained for free quercetin and quercetin-loaded SA-PVA hydrogels. **** *p* < 0.0001 for all hydrogel formulations vs. free quercetin at 24 h; # *p* < 0.05 for 1/1 vs. 1/2 hydrogel formulation and &&&& *p* < 0.0001 for 2/1 vs. 1/2 hydrogel formulation both at 24 h. (**C**) Distribution of quercetin among permeated, retained, and non-permeated through the PVPA*_SC_* after 24 h. The bars/points represent the mean ± SD of the permeability for at least three independent experiments (*n* = 3). * *p* < 0.05 for 2/1 hydrogel formulation vs. other formulations and free quercetin remained in apical medium.

The apparent permeability data (*P_app_*) at 3 h for quercetin-loaded hydrogels indicated a similar permeation rate for all the studied formulations as well as for the free drug (Figure 7A). Free quercetin showed a higher permeation rate thus being less in contact with the model membrane (Figure 7B) while the 2/1 combination presented a low permeation rate (*p* > 0.05). Similar to the results obtained from the isolated *SC* model (Figure S2), the permeation of the free quercetin and drug-loaded hydrogels increased along the time up to 24 h. However, a significantly higher permeation (maximum of 19% after 24 h) was found for free quercetin than for all hydrogel formulations at the considered timepoints (*p* < 0.05–0.0001) except at 1 h when no significant differences were determined. At the later timepoint it was possible to recognize the distinct permeation profile of the various hydrogel formulations, particularly for 1/2 formulation which permeated in a higher percentage (12%) than 1/1 or 2/1 combinations (*p* < 0.05) (Figure 7C). Quercetin remaining in the donor compartment corresponded to 30 (for the free form) to 71% of the initial amount of drug, being the 2/1 SA-PVA hydrogel, the formulation presenting higher percentage of quercetin in that compartment and therefore yet available to permeate within the skin. About 187.6 ± 92.7, 216.9 ± 56.5, and 213.6 ± 96.1 µg/cm^2^ of quercetin-loaded in the 1/1, 2/1, and 1/2 SA-PVA hydrogels, respectively remained in the PVPA*_SC_* barrier, similar to the amount determined for free quercetin, 276.8 ± 63.2 µg/cm^2^ after 24 h of contact (Figure 7C). Generally, 1/1 and 1/2 combinations revealed a similar penetration/permeation profile.

Taken together, the results obtained from both skin mimetic models revealed a similar high penetration and low permeation profile for the quercetin-loaded 2/1 SA-PVA hydrogels after 24 h of contact. In fact, permeation of antioxidants such as quercetin is undesirable, as dissemination into the systemic circulation will decrease photoprotection and local inflammation control [19]. Achieving control of skin inflammatory conditions or dermo-cosmetic applications requires accumulation of the bioactive compound in the *SC* and viable epidermis layers. Hydrogels with different compositions have been designed in order to enhance quercetin skin accumulation with low transepidermal permeation using carbomer [20], carbopol [21], or chitosan [15]. Data described in these publications have a similar profile to what was found for SA-PVA hydrogels, yet no direct correlation can be made as different skin models and permeation assay conditions were used.

The overall results pointed out that quercetin-loaded hydrogel formulations may present an advantage over the administration of the flavonoid dissolved in dissolving agents such as miglyol, by decreasing the permeation rate and increasing the penetration and time of interaction of the drug within the skin, thus increasing their local effect and decreasing the eventual toxicity of those types of solvents.

## 4. Conclusions

The present work establishes the designed SA-PVA hybrid hydrogels as effective delivery systems for bioactive polyphenols intended for pharmaceutical applications. Blends of different ratios of SA and PVA biopolymers successfully produced three types of hybrid hydrogels, able to incorporate quercetin within the polymeric network. Rheological analysis revealed robust systems with pseudoplastic behavior, a non-thixotropic profile, and good resistance to deformation and temperatures from 20 to 40 °C, making them good candidates for cutaneous application. All the hybrid hydrogels retained quercetin antioxidant activity and promoted the entry of quercetin in the skin. Ex vivo studies of quercetin accumulation and permeation through the *SC* from a hydrogel formulation underlined a certain role of the biopolymers in hindering quercetin permeation. Despite the similar performance and characteristics of all the designed hydrogels, the blend 2/1 seems to cover most of the desirable features to be used in the treatment of skin conditions associated with oxidative stress and inflammation. The 2/1 quercetin-loaded SA-PVA hydrogel presents the highest swelling and viscosity profile as well as the lowest permeation and a highest penetration rate on the skin mimetic models, contributing to a more effective local skin effect. These hybrid hydrogels can represent a promising platform for the delivery of flavonoids for skin therapeutic applications.

## Supplementary Materials

The following are available online at https://www.mdpi.com/1999-4923/12/12/1149/s1, Figure S1: Resistance to temperature ramp from 20 to 40 °C of unloaded (top line) and quercetin-loaded (bottom line) hydrogels; Figure S2: Quercetin permeation profile through the isolated *SC* layer.
