# Supplementary material for: Design and Characterization of Sodium Alginate and Poly(vinyl) Alcohol Hydrogels for Enhanced Skin Delivery of Quercetin
Source: Pharmaceutics. 2020 Nov 27;12(12):1149. doi: 10.3390/pharmaceutics12121149 (PMC7760628; doi:10.3390/pharmaceutics12121149)
Supplement: Supplementary file 1 [file pharmaceutics-12-01149-s001.pdf]

pharmaceutics

Supplementary Materials: Design and Characterization of Sodium Alginate and Poly(vinyl) Alcohol Hydrogels for Enhanced Skin Delivery of Quercetin

Ludovico Esposito, Ana Isabel Barbosa, Tânia Moniz, Sofia Costa Lima, Paulo Costa, Christian Celia and Salette Reis

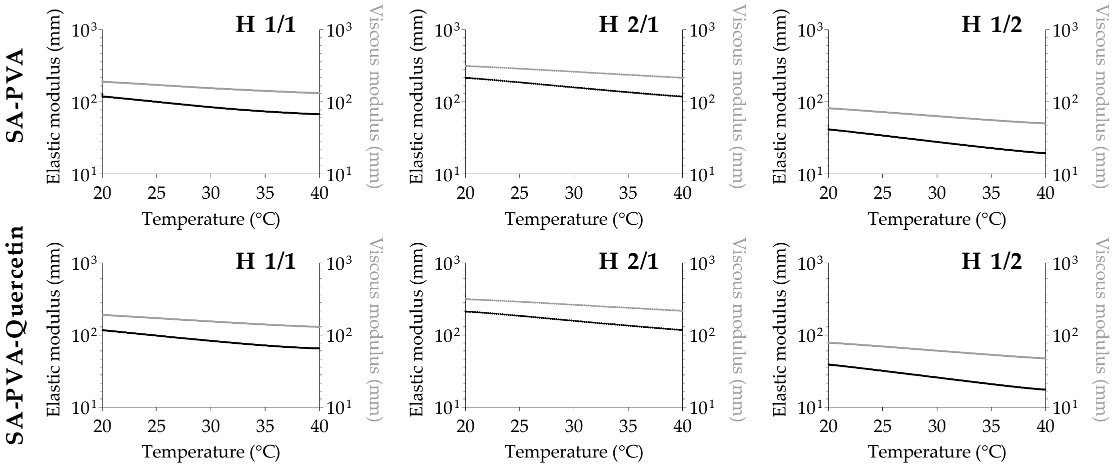

Figure S1. Resistance to temperature ramp from 20 to 40 °C of unloaded (top line) and quercetin-loaded (bottom line) hydrogels.

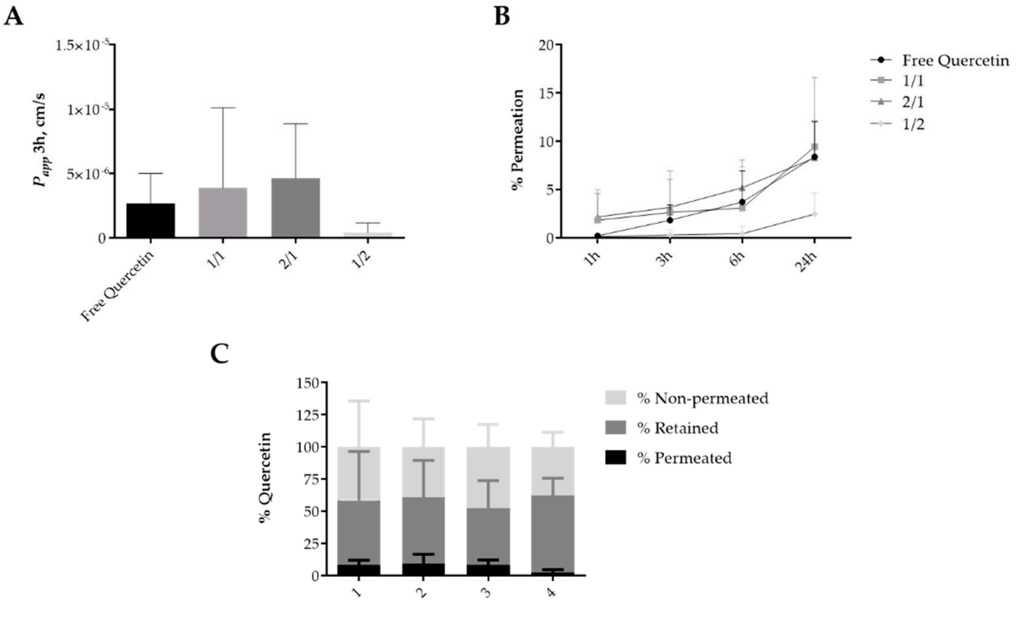

Figure S2. Quercetin permeation profile through the isolated SC layer. (A) Apparent permeability (P_{app}) of quercetin at 3 h; (B) Amount of permeated quercetin (%) as a function of time obtained for free quercetin and quercetin-loaded SA-PVA hydrogels. (C) Distribution of quercetin among permeated, retained and non-permeated through the *SC* after 24 h. The bars/points represent the mean ± SD of the permeability for at least three independent experiments ($n = 3$).
